# A Questionnaire-Based Survey to Assess the Level of Knowledge and Awareness about Drug–Food Interactions among General Public in Western Saudi Arabia

**DOI:** 10.3390/pharmacy9020076

**Published:** 2021-04-08

**Authors:** Syed Faisal Zaidi, Rayan Mgarry, Abdullah Alsanea, Sakar Khalid Almutairi, Yaser Alsinnari, Saad Alsobaei, Kanwal Ahmed

**Affiliations:** 1 College of Medicine, King Saud bin Abdulaziz University for Health Science, Jeddah 21423, Saudi Arabia; mgarryr@merrimack.edu (R.M.); alsaneaa@merrimack.edu (A.A.); almutairis@merrimack.edu (S.K.A.); alsinnariy@merrimack.edu (Y.A.); alsobaeis@merrimack.edu (S.A.); kanwalahd@gmail.com (K.A.); 2 Department of Pharmacology, School of Medicine, Batterjee Medical College for Sciences and Technology, Jeddah 21442, Saudi Arabia; 3 Fakeeh College for Medical Sciences, Jeddah 21461, Saudi Arabia

**Keywords:** drug–food interaction, Kingdom of Saudi Arabia, knowledge, awareness, general public, Jeddah

## Abstract

**Introduction:** Various drug–food interactions exist that may hinder treatment and can sometimes be lethal. Our aim was to assess the level of public knowledge and awareness in Jeddah city, Western Saudi Arabia, about drug–food interactions, along with the effects of demographics on their knowledge. **Methods:** A survey questionnaire was administered in this cross-sectional study to participants spread across multiple locations in Jeddah, including in malls and public gatherings. Participants included both males and females. Sample size was calculated through Raosoft^®^ software. Data analysis was executed using IBM Statistic SPSS and the level of statistical significance was set at *p* < 0.05. **Results:** A total of 410 people participated in the study and only 92.68% (380) of responses were enrolled in the study; 7.32% (30) were not enrolled due to the exclusion criteria. Surprisingly, only six out of eighteen questions regarding drug–food interactions in the administered questionnaire were correctly answered by 380 participants. Data indicated that the participants had a poor to intermediate level of both knowledge and awareness with respect to drug–food interactions. Furthermore, participants showed moderate to strong awareness of the effects of alcohol and tea generally, and their interaction with medication. **Conclusion:** Participants in our study showed inadequate knowledge of basic and fundamental information about drug–food interactions, which highlights the dire need to increase awareness.

## 1. Introduction

Research has shown that some foods, particularly vegetables and fruits, can interact with the pharmacokinetics and pharmacodynamics of some drugs [1]. In a study which reviewed the original literature, Schmidt and Dalhoff tried to explain the clinical relevance of drug–food interactions and stated that the most important interactions are the ones that hinder the treatment plan by reducing the bioavailability of the drug [1]. They gave an example of taking the antibiotic ciprofloxacin with dairy, which reduces the amount of antibiotic reaching the bloodstream and interferes with the course of treatment. Schmidt and Dalhoff also added that increasing awareness in the United States about drug–food interactions would assist in reducing drug side effects. Another classic example of drug-food interaction (DFI) concerns grapefruit, which inhibits Cytochrome P450 (CYP3A4), the most important enzyme in drug metabolism, located in the mitochondrial electron transport chain, and consequently increases the bioavailability of cyclosporine, which increases the drug’s toxicity [2]. Another study was conducted in Washington, DC, USA to assess the knowledge, attitudes and awareness of drug and food interactions among nurses of different levels of knowledge and experience. The disparity among the groups of nurses was significant, and 72.3% of nurses had not observed drug–food interactions in the healthcare setting. Training on DFI every 6 months was recommended for the participants [3].

Examples of drug–food interaction include grapefruit juice with pirfenidone, a drug that was administered to treat idiopathic pulmonary fibrosis (IPF) in a randomized control trial in China. The results showed a severe reduction in peak concentrations of the drug in those who took the medication with grape juice compared to the control group [4]. In addition, taking the anti-coagulant agent warfarin with a high-protein diet can lead to increased serum albumin concentration and possibly result in increased blood coagulability (thickness) and increased risk of blood clot formation [5]. Green vegetables such as spinach and broccoli are great examples of vitamin-K-rich sources. Vitamin K is an essential blood clotting factor to prevent bleeding. In patients undergoing anticoagulant therapy, such as warfarin to prevent thromboembolism, these vitamin K green vegetables have been shown to interact with warfarin [6]. Although consumption of these vitamin K sources moderately increases international normalized ratio (INR) linearly and does not affect dose requirement, consumption of large quantities may hinder the effectiveness and safety of warfarin therapy [6,7]. It is important to keep in mind that, although these green vegetables are high in vitamin K, the relative bioavailability is only 13% and 29% for broccoli and spinach, respectively [8]. Thus, consuming a moderate amount will not affect therapy but consuming large quantities on a regular basis is not recommended when undergoing anticoagulant therapy. An example of severe DFI involves fermented foods such as cheese and wine, which contain tyramine, which interacts with monoamine oxidase inhibitors (MAOIs, a class of drugs used to treat depression). MAOIs inhibit the breakdown of tyramine, causing buildup of catecholamine (vasoconstrictor), resulting in life-threatening hypertensive crisis [9,10]

In the UAE, a study was conducted to assess the knowledge of drug–drug, drug–disease, and drug–food interactions, and it emphasized the importance of drug–food interactions and showed poor public awareness [11]. Most participants demonstrated good knowledge regarding drug interaction types, with correct response rates of 72% and 69% for males and females, respectively. Although participants showed good knowledge of drug interaction types, the results illustrated that most participants had poor awareness of over-the-counter medication (OTC) and prescribed medication interactions with herbal remedies and supplements, with correct responses of 60.4%. Interestingly, their lowest correct response rate was that of age group >40 in every section of their questionnaire [11]. Another questionnaire-based study in Al-Madinah Al-Munawarah was conducted to assess the knowledge of healthcare professionals on adverse drug reactions and drug safety. The study reported that healthcare professionals had good knowledge of adverse drug reactions but poor knowledge about drug safety (pharmacovigilance) [12]. The study also showed that there were no adverse drug reaction (ADR) reporting and pharmacovigilance systems implemented in hospitals. To the best of our knowledge, only one questionnaire-based study from Jeddah has been reported to assess public knowledge of food–food interactions and drug–food interactions [13]. The results showed that 62.1% of respondents lacked knowledge about food–food and drug–food interactions that affect nutritional status. The study’s shortcoming was that it focused on food–food interactions rather than drug–food interactions; however, we generally lack research studies on both aspects in the Kingdom of Saudi Arabia and around the Gulf area. 

Therefore, this survey study was conducted to assess the level of knowledge and awareness of people in Jeddah, KSA regarding DFI. Moreover, it is designed to identify any correlations between demographic data, i.e., gender, age, and the level of knowledge and awareness of taking drugs with food.

## 2. Materials and Methods

### 2.1. Study Design, Area, and Settings

The study was a descriptive observational cross-sectional design and was conducted between January and May, 2019. Data gathering took place across multiple locations in Jeddah, including the Mall of Arabia in the Center of Jeddah, Red Sea Mall in the North of Jeddah, Al Yasmin Mall in East Jeddah, Andalus Mall in the South of Jeddah, and Jeddah Waterfront on the West Coast of Jeddah. Attendees at these places represent different socioeconomic and educational statuses. The questionnaire was printed on A4 paper and handed to participants by medical students from King Saud bin Abdulaziz University, Jeddah. The participants filled out the questionnaires in the presence of medical students, who answered any questions and then collected the papers from participants.

### 2.2. Study Approval

The study was approved by the Institutional Review Board (IRB) of King Abdullah International Medical Research Center, Jeddah, Saudi Arabia with IRB approval number SP18/337/J. Informed consent was obtained from each participant before data collection.

### 2.3. Study Participants and Sample Size

The study included both genders, with a minimum age of eighteen years old, with at least a high school diploma. Sample size was calculated using the Raosoft^®^ software at the developer’s website. The required sample size was estimated at 95 percent confidence level with an estimated 50% response distribution and a margin of error of ±5%. The required minimum sample size was determined to be 384; the final sample size was 410 to account for a 10% non-response rate [14].

### 2.4. Data Collection Process

This study utilized a self-administered questionnaire consisting of 33 questions adapted from previous studies, including multiple-choice questions [15,16]. The questionnaire was validated by conducting face validity with the aid of experts in the Department of Medical Education at King Saud bin Abdulaziz University of Health Sciences, Jeddah. Pilot study was carried out to check the clarity of the questions. Modifications were made to the questionnaire according to the feedback from the pilot testing. The linguistic expert checked the Arabic translation. The completed questionnaires were collected in person by data collectors and were then processed using Microsoft Excel for analysis.

Non-probability convenience sampling technique was used for selecting participants. Our material was a survey questionnaire that the public was provided with at designated sites and within a specific period. The questionnaire consisted of three parts of descriptive data, plus a demographic part. Part one included general questions about the use of medications and antibiotics. Part two involved the assessment of knowledge, using questions regarding the participants’ knowledge of drug–food interactions. Part three included general drug–food interaction questions to assess awareness. The questionnaires were printed and handed to willing participants. Thereafter, we collected the data and entered them into the SPSS program.

### 2.5. Score Calculation

To assess the knowledge and awareness, there were a total of 18 questions in this study: 12 questions to assess the knowledge, and 6 questions to assess the awareness. The knowledge assessment was based on “yes/no/I don’t know” questions, where “yes” had a score of 1, and “no/I don’t know” had a score of 0. The awareness assessment was based on multiple-choice questions, where the correct answer had a score of 1, and the other answers had a score of 0. Table 1 describes the grading of participants according to the level of knowledge.

### 2.6. Data Analysis

Data analysis was conducted using IBM SPSS Statistics (SPSS Inc., Chicago, IL, USA) Version 26 that was downloaded from the IBM official website. Descriptive analysis was employed to sum up the dataset, e.g., demographic characteristics, knowledge, and awareness of DFI. To test the influence of demographic characteristics on knowledge and awareness, the chi-square test was applied wherever suitable, and *p*-values were obtained. The level of statistical significance was set at *p* < 0.05. The knowledge and awareness of the participants was assessed based on the answers to the questionnaire.

## 3. Results

A total of 410 people participated in the study; 92.68% (380) of responses were enrolled in the study and 7.32% (30) were not enrolled due to the exclusion criteria (<age of 18 and <high school degree). People who participated, based on gender, were 52.4% (199) male and 47.6% (181) female. Regarding age, the average was 36 and most of the participants were between the ages of 35 and 44 years old, accounting for 25.0% (95 out of 380). Most of the participants had a college degree, namely 67.4% (256). A total of 80% did not have an occupation related to healthcare, and 60.3% of participants’ family members did not have an occupation related to healthcare either. In terms of whether the participants had a chronic disease or not, a total of 66.6% (253) did not have any chronic diseases. All these results are listed in Table 2 (demographics). 

Out of the total 18 questions that assessed knowledge and awareness, only six questions were answered correctly (had a response rate of 60% or greater out of the total participants), namely four questions in the knowledge assessment part, and two in the awareness assessment part. (Table 3). A chi-square test was conducted on these six questions to identify a significant relationship with demographic characteristics (age, gender, education level, occupation related to healthcare, family occupation related to healthcare, and chronic diseases). Significant *p*-values of 0.002 and 0.015 were found between question (C2), which assessed the need to know about drug–food interaction and gender (female) and chronic diseases, respectively. Question (C4), which asked about the effect of food combinations on medication efficacy, had a significant *p*-value of 0.005 with occupation related to healthcare (participants who answered yes). Question (C6), which assessed various factors such as drug dose, age, and health status and their impact on drug–food interaction, had a significant relationship with gender (female) and family occupation related to healthcare (participants who answered yes), with *p*-values of 0.032 and 0.020, respectively. Question (C9), which asked if all drugs can be taken with food, had a significant *p*-value of 0.014 with gender (female). Question (D2), which assessed the knowledge of avoiding the consumption of certain beverages when taking medications, had a *p*-value of 0.021 with occupation related to healthcare (participants who answered yes). Question (D6), which assessed the iron supplement absorption rate when consumed with certain beverages, had *p*-values of 0.010 and 0.001 with age (> 54 Y/O) and gender (female), respectively (Table 4).

## 4. Discussion

Drug–food interactions are of great importance in delivering effective drug therapy. Adverse effects of drug–food interaction reduce the bioavailability of drugs, render therapy regimens ineffective, and can sometimes be lethal. Prescribing the right drug for the right patient at the right dose is safest, but an interaction with food consumed while on drug therapy can interfere with the treatment [17]. This will not only result in failure of treatment but also lead to higher healthcare costs. As such, our research aim in this study was to assess the general public’s knowledge on this subject, to avoid such negative effects and alleviate the healthcare cost burden.

In the assessment of the level of knowledge, Table 3 shows that only one third of the general knowledge survey questions were answered correctly by the majority of participants (4 out of 12 total questions: majority equal to 60% and greater). Table 3 also shows that most participants ranged between moderate to good in the assessment of level of knowledge, specifically for the first basic questions (C1 to C9). These results suggest that the participants have moderate to good basic knowledge about drug–food interactions, and they recognize the importance of this topic.

However, the participants failed to correctly answer the more complex questions about their level of knowledge. When asked about whether all drugs can be taken on an empty stomach to produce better effects (C10), only 5% of the participants (19 out of 380) correctly answered this question, which implies a lack of knowledge regarding the timing of medication administration. Medications such as carvedilol are instructed to be taken with food to avoid orthostatic hypotension [18]. Meanwhile, levothyroxine, a treatment for thyroid dysfunction, is advised to be taken on an empty stomach to maximize drug absorption and effect [18,19]. Moreover, when asked about whether acidic food and beverages can be taken with antibiotics, only 12.9% of the participants (49 out of 380) correctly answered this question. These results suggest a poor level of knowledge regarding the more sophisticated, yet important, information that the public should be aware of regarding about drug–food interactions.

In the assessment of awareness level among participants, only two questions out of six were answered correctly. Though these questions are specific and may require clinical knowledge in order to answer correctly, the participants were able to correctly answer the questions related to “avoid drinking alcohol when taking medications” (question D2, 65% correct) and “drinking tea reduces iron body absorption” (question D6, 71.8% correct). These results suggest that the participants are aware of the side effects of alcohol and its interaction with drugs in general. This may be correlated to the Islamic beliefs that alcohol is harmful, and it is banned in Saudi Arabia. For question D6, the participants successfully chose the correct option, and this suggests a good level of awareness that drinking tea may reduce the effects of iron supplements and iron absorption by the body [20,21]. This can be correlated to the tradition in Saudi Arabia of serving tea after meals, and since iron deficiency anemia is common in the region, participants could have answered this correctly because of how common iron deficiency anemia is and the long-standing tradition of tea-drinking [22].

Similar to our study, a study that was previously conducted in Jeddah, by Abualhamail et al., concluded in 2016 that “nutritional knowledge is poor among Saudis, with folklore and media being the main sources of information” [13]. Though the study by Abualhamail et al. focused on food–food interactions, some aspects of the two surveys were similar. The question regarding iron absorption and tea drinking interaction was asked in Abulhamail et al.; however, the knowledge was poor, and the answer was not known by 63% of the sample (N = 998) [12]. Moreover, the percentage was even higher for participants with less than high school education. In Ajman in the United Arab Emirates, a public awareness campaign that has been conducted was focused only drug–drug interactions [11]. This study concluded that most of the participants were unaware of possible interactions between prescribed and over-the-counter (OTC) drugs or even with herbal medications and supplements. The significant value regarding possible interactions between prescribed drugs, (OTC), herbal medications, and supplements was (*p* < 0.01), which suggests similarity in the poor public knowledge regarding the usage of medications [11].

Poor knowledge of DFI is not only found in the general public, but it is also found among healthcare workers, as some studies suggest. In this regard, a study was conducted to assess knowledge of DFI and DDI among professors, postgraduates, and interns in a tertiary care hospital in Mysore, India [15]. Results showed that while professors had a good understanding of DFI, interns and postgraduates did not [15]. Even when some healthcare workers are aware of certain DFIs, such as those involving vitamin-K-containing foods and anticoagulants, most were not aware of interactions present between dairy products with tetracycline and fluoroquinolones, which cause the formation of chelate and reduce its bioavailability [15,23]. The study in India also concluded that postgraduates and interns’ knowledge was not adequate and only a third of healthcare workers recorded DFI during clinical practice [15]. This correlates with our findings that while the public had a good understanding of some DFI interactions, they were unaware of other important interactions. This also extends to some healthcare workers, especially at the junior levels, as some studies suggested. As such, it is recommended that healthcare workers, especially interns who have recently started their clinical practice, be educated and regularly exposed to DFI and their interactions [24].

Another study was done by distributing questionnaires online through social media to compare knowledge of DFI in members of the general public from diverse backgrounds [16]. The study illustrated that clinical pharmacologists had better knowledge of DFI than doctors, nurses, and pharmacy students. Conversely, those of non-medical background demonstrated even less DFI knowledge than those with a medical background [16]. The survey also found that the general public had been using herbal supplements concomitantly with prescription and over-the-counter medication because of a lack of knowledge [16]. The study also encouraged training and integration of DFI knowledge among healthcare workers to reduce such potentially harmful practices [16]. We strongly concur, as empowering healthcare workers with knowledge and awareness will also impact the general public’s knowledge positively.

Some limitations were found and identified while conducting our study. The sample size was relatively low, and if the sample size was larger, the results would suggest stronger correlations between the different variables. Moreover, the design was cross-sectional, which could be affected by the time because the surveys were done at a certain point in time. Lastly, the data that we collected in this study were only related to one city in Saudi Arabia, namely Jeddah. Inclusion of more cities, or at least neighboring towns and cities, such as Makkah and Taif, would produce a better understanding of the Western Region of Saudi Arabia as a whole.

We suggest further wide evaluation of knowledge and awareness around the Kingdom to assess the population’s general knowledge regarding drug–food interactions. In addition, further awareness campaigns should be conducted to increase the public’s knowledge, especially if future research yields similar results to ours, where the majority of the general public is unaware of drug–food interactions and lacks knowledge in this matter. Importantly, the impact of these awareness campaigns on the public’s knowledge regarding DFI should be critically analyzed for future directions. Moreover, a survey assessing healthcare workers’ knowledge of DFI in the same demographic area should be conducted to correlate the association of DFI public knowledge with healthcare workers.

## 5. Conclusions

Participants from Jeddah in our study showed moderate knowledge of basic and fundamental aspects of drug–food interactions. Moreover, participants showed moderate to good awareness of the effects of alcohol and tea generally, and their interaction with medication. In addition, the results from the study may help physicians in the better management of certain diseases where drug–food interaction is crucial, i.e., prescribing the appropriate drug and dose, taking into consideration the patient’s dietary needs and choices. Other than physicians, the results of this study may also help to raise the awareness of the general public regarding drug–food interactions, even by simply asking them questions in this regard, which may encourage them to think about the interactions and the side effects of combining certain drugs with certain foods. Overall, there is still poor knowledge and awareness among people in Jeddah, with an inadequate understanding of drug–food interactions.

## Figures and Tables

**Table 1:** Grading of participants’ level of knowledge and awareness.

Level of Knowledge/Awareness	Correct Answer (Out of 18 Questions)
Poor	<30%
Moderate	31%–59%
Good	≥60%

**Table 2:** Demographic characteristics based on total number of participants.

Demographics		Total Number of Participants (N = 380)	Percentage %
Age	18–24 Y/O	90	23.7
	25–34 Y/O	89	23.4
	35–44 Y/O	95	25.0
	45–54 Y/O	78	20.5
	> 54 Y/O	28	7.4
Gender	Male	199	52.4
	Female	181	47.6
Education Level	High School	99	26.1
	College	256	67.4
	Higher education	25	6.6
Occupation Related to Healthcare	Yes	76	20
	No	304	80
Family Member’s Occupation Related to Healthcare	Yes	151	39.7
	No	229	60.3
Chronic Disease	Yes	127	33.4
	No	253	66.6

**Table 3:** Level of knowledge and awareness based on correct answers for each question. (e.g., Question C1, 121 participants out of 380 answered correctly, which equates to 31.8% (moderate level, see Table 2) of participants).

Questions		Level of Knowledge and Awareness among Participants Based on Correct Answers		
		Poor <30%	Moderate 31%–59%	Good ≥60%
Knowledge (C1–12)	(C1) Do you have knowledge about drug-food interaction?	-	(121) 31.8%	-
	(C2) It is necessary to know about the Drug-Food Interaction?	-	-	(316) 83.2%
	(C3) Over the counter (OTC) and prescription medicines do not interact with food?	-	(128) 33.7%	-
	(C4) Do you think that food combinations can affect the efficacy of medications?	-	-	(254) 66.8%
	(C5) Do you know that food can speed up or slow down the action of a drug?	-	(219) 57.6%	-
	(C6) Is the Impact of Drug-Food Interaction depending on a various factor like drug dosage, person’s age, & health status?	-	-	(261) 68.7%
	(C7) Drug-Food Interaction can lead to serious side effects?	-	(210) 55.3%	-
	(C8) Do you think Drug-Food Interaction can cause death?	-	(139) 36.6%	-
	(C9) All drugs can be taken with food?	-	-	(236) 62.1%
	(C10) All the drugs can be taken on an empty stomach to produce better effects?	(19) 5.0%	-	-
	(C11) Is it better to avoid taking milk & dairy products, iron–rich food and supplements with certain antibiotics?	(103) 27.1%	-	-
	(C12) Acidic foods and beverages-such as tomato sauce, tea, coffee, and citrus juices can be taken along with antibiotics?	(49) 12.9%	-	-
Awareness (D1–6)	(D1) Which age group of patients do you think are at a greater risk of developing drug-food interaction?	(64) 16.8%	-	-
	(D2) Which of the following beverages do health experts recommend you avoid when taking medications?	-	-	(247) 65%
	(D3) This fruit interacts with around 45 different medicines and produces lethal side effects?	(71) 18.7%	-	-
	(D4) Asthma medications should not be taken with?	(70) 18.4%	-	-
	(D5) Patients on drugs for diabetes should avoid?	-	(157) 41.3%	-
	(D6) Iron supplements has its absorption reduced by?	-	-	(273) 71.8%

**Table 4:** Association of demographic characteristics with level of knowledge and awareness of ≥60%.

Question #	Question/Statement	Correct Answer (N = 380)	*p*-Value (Pearson Chi-Square Test)					
			Age	Gender	Education Level	Occupation Related to Healthcare	Family Occupation Related to Healthcare	Chronic Diseases
C2	It is necessary to know about the Drug-Food Interaction	316 (83.2%)	0.217	0.002	0.657	0.193	0.072	0.015
C4	Do you think that food combinations can affect the efficacy of medications?	254 (66.8%)	0.075	0.511	0.884	0.005	0.177	0.369
C6	Is the Impact of Drug-Food Interaction depending on a various factor like drug dosage, person’s age, & health status?	261 (68.7%)	0.385	0.032	0.079	0.184	0.020	0.263
C9	All drugs can be taken with food	236 (62.1%)	0.140	0.014	0.794	0.072	0.362	0.355
D2	Which of the following beverages do health experts recommend you avoid when taking medications	247 (65.0%)	0.184	0.062	0.454	0.021	0.531	0.576
D6	Iron supplements has its absorption reduced by	273 (71.8%)	0.010	0.001	0.592	0.332	0.129	0.504

## Data Availability

The datasets generated or analyzed during the study are available from the corresponding author on acceptable request.
